# Diffusion of smoke-free policies at outdoor sports clubs in the Netherlands

**DOI:** 10.1093/eurpub/ckac129.753

**Published:** 2022-10-25

**Authors:** HH Garritsen, AE Kunst

**Affiliations:** Department of Public and Occupational Health, Amsterdam UMC, University of Amsterdam, Amsterdam, Netherlands

## Abstract

**Background:**

Although outdoor smoke-free policies (SFPs) at sports clubs represent an important new area of tobacco control, the majority of sports clubs are not smoke-free. This study aims to assess diffusion patterns of outdoor SFPs at sports clubs in the Netherlands, which may inform national strategies aimed at making all outdoor sports clubs smoke-free.

**Methods:**

Using a retrospective, registry-based design, an inventory was made of football, field hockey, tennis, and korfball clubs that became smoke-free between 2016-2020. We determined the type of sports, number of members, and proportion of youth members. The degree of urbanization and density of smoke-free sports clubs were measured at the municipality level. The association between sports clubs’ characteristics, degree of urbanization, and SFP adoption was analysed using multilevel regression analysis. Horizontal diffusion was tested by analysing the association between the density and annual incidence of smoke-free sports clubs.

**Results:**

Since 2016, the number of sports clubs with an outdoor SFP increased from 0.3% to 26.4%. Field hockey [OR compared to football 6.00 95% CI 4.46-8.07] and korfball clubs [OR 6.65 95% CI 4.98-8.87] and clubs with many (youth) members [OR 8.75 95% CI 6.20-2.35] were more likely to be smoke-free. SFPs spread from the most urbanized to less urbanized municipalities, which could mostly be attributed to sports clubs’ characteristics. A higher density of smoke-free sports clubs within municipalities was associated with an increased incidence of new SFPs in the following year.

**Conclusions:**

Outdoor SFPs at sports clubs in the Netherlands diffused across horizontal and hierarchical lines. National strategies for smoke-free sports should monitor clubs that are more likely to stay behind, such as football and tennis clubs, smaller clubs, and clubs in less urbanized areas.

